# A Low Complexity Rapid Molecular Method for Detection of *Clostridium difficile* in Stool

**DOI:** 10.1371/journal.pone.0083808

**Published:** 2014-01-08

**Authors:** Cathal J. McElgunn, Clint R. Pereira, Nicholas J. Parham, James E. Smythe, Michael J. Wigglesworth, Anna Smielewska, Surendra A. Parmar, Olga A. Gandelman, Nicholas M. Brown, Laurence C. Tisi, Martin D. Curran

**Affiliations:** 1 Lumora Ltd, Ely, Cambridgeshire, United Kingdom; 2 Public Health England, Clinical Microbiology and Public Health Laboratory, Addenbrooke's Hospital, Cambridge, Cambridgeshire, United Kingdom; Cornell University, United States of America

## Abstract

Here we describe a method for the detection of *Clostridium difficile* from stool using a novel low-complexity and rapid extraction process called Heat Elution (HE). The HE method is two-step and takes just 10 minutes, no specialist instruments are required and there is minimal hands-on time. A test method using HE was developed in conjunction with Loop-mediated Isothermal Amplification (LAMP) combined with the real-time bioluminescent reporter system known as BART targeting the toxin B gene (*tcdB*). The HE-LAMP-BART method was evaluated in a pilot study on clinical fecal samples (*tcdB* ^+^, n =  111; *tcdB* ^−^, n  = 107). The HE-LAMP-BART method showed 95.5% sensitivity and 100% specificity against a gold standard reference method using cytotoxigenic culture and also a silica-based robotic extraction followed by *tcdB* PCR to control for storage. From sample to result, the HE-LAMP-BART method typically took 50 minutes, whereas the PCR method took >2.5 hours.

In a further study (*tcdB* ^+^, n =  47; *tcdB* ^−^, n  = 28) HE-LAMP-BART was compared to an alternative commercially available LAMP-based method, Illumigene (Meridian Bioscience, OH), and yielded 87.2% sensitivity and 100% specificity for the HE-LAMP-BART method compared to 76.6% and 100%, respectively, for Illumigene against the reference method. A subset of 27 samples (*tcdB* ^+^, n =  25; *tcdB* ^−^, n  = 2) were further compared between HE-LAMP-BART, Illumigene, GeneXpert (Cepheid, Sunnyvale, CA) and RIDA®QUICK *C. difficile* Toxin A/B lateral flow rapid test (R-Biopharm, Darmstadt, Germany) resulting in sensitivities of HE-LAMP-BART 92%, Illumigene 72% GeneXpert 96% and RIDAQuick 76% against the reference method. The HE-LAMP-BART method offers the advantages of molecular based approaches without the cost and complexity usually associated with molecular tests. Further, the rapid time-to-result and simple protocol means the method can be applied away from the centralized laboratory settings.

## Introduction

*Clostridium difficil*e is a Gram positive bacterium that is the most frequent cause of infectious bacterial diarrhea worldwide [1]. *C. difficile* infection (CDI), which is associated with the use of broad spectrum antibiotics to treat other underlying conditions, results in the release of the two main virulence factors, toxins A and B [2], that result in mild to severe watery diarrhea with CDI [3]–[4].

An epidemic of CDI with continuously increasing rates was seen in the USA, Canada and most of Europe starting around 2000–2002. This epidemic was mostly associated with the hypervirulent ribotype 027/NAP1 B1 strain. This renewed interest in CDI towards pathophysiology, prevention, detection and treatment [5]–[7]. In England, voluntary surveillance showed an increase in CDI from 1990 to a peak of 52,983 reports in 2007. However, following successful implementation of control measures, there were 13,352 reports in 2012, a reduction of 75% [8]. Mandatory surveillance was introduced in April 2007 and confirms this fall in cases. This may be due to the emergence and subsequent decline of hypervirulent strains of *C. difficile* (*e.g.*, ribotype 027/NAP1, responsible for 55% of isolates ribotyped in 2007/08, but only 12% of isolates in 2010/11) [9]. The 027/NAP1 strain has an expanded repertoire of antibiotic resistance elements, produces greater quantities of toxins, and therefore gives rise to more severe disease with higher mortality rates and an increased probability of relapse following clinical treatment [10]–[11].

Laboratory diagnosis of CDI from stool has traditionally been based on detecting the presence of *C. difficile* toxin A and/or toxin B proteins in stool by various methods including cytotoxicity assays and Enzyme Immuno Assay (EIA) [12]–[15]. In general, these methods should meet the minimum desirable characteristics of a diagnostic test of sensitivity, specificity, cost-efficiency, rapid results, ease of use preferably by non-expert users and ability to differentiate between toxigenic and non-toxigenic *C. difficile*. Cell cytotoxicity assays are sensitive, but are extremely time-consuming and their requirement for cell-culture prohibits their application for near-patient rapid testing. EIA tests for one or both toxins are relatively insensitive, detecting only 30% to 70% of CDI-related disease [16]–[20]. Moreover, toxin gene expression is known to be repressed in nutrient rich conditions [21]. With the development of an EIA for glutamate dehydrogenase (GDH), which is a *C. difficile* cell wall common antigen, the sensitivity for the detection of *C. difficile* approaches 100%; however, because GDH is ubiquitous for both toxigenic and non-toxigenic strains, specificity of the GDH assay is poor [11]. Therefore, individual EIA tests for either GDH or toxins A or B are considered to be insufficiently specific for diagnosis. However, the combination of GDH and toxin A/B, such as in the *C. DIFF* QUIK CHEK COMPLETE EIA assay (Techlab, Blacksburg, VA), gives increased diagnostic sensitivity and specificity provided the results for both analytes are concordant [14]–[15]. However, when GDH and toxin results are discordant, additional reflex or discrepant testing using a highly specific molecular-based assay is required [22].

Molecular-based assays for the detection of toxigenic *C. difficile* in stool offer increased sensitivity over and are as specific as EIA tests [16], [23]. Genes within the *C. difficile* pathogenicity locus, including those encoding toxins A and/or B (*tcdA* or *tcdB*), have been targeted by specific primer sets, usually in a multiplex PCR assay. Identification of these gene sequences in stool samples correlates highly with positive *C. difficile* toxigenic stool assays [17]. A positive PCR assay is therefore thought to directly indicate the presence of toxigenic *C. difficile* strains in stool [24]–[26]. Accordingly, new molecular assays such as the Loop-mediated Isothermal Amplification (LAMP) assay (Illumigene *C. difficile*; Meridian Bioscience, Cincinnati, OH) and the GeneXpert assay (Xpert *C. difficile*; Cepheid, Sunnyvale, CA), which detect pathogenicity locus region sequences, are being increasingly used for confirmatory testing of EIA tests. Nonetheless, despite greater diagnostic sensitivity and specificity, current molecular methods are too labor-intensive, complex, and/or costly to be of practical use in many clinical laboratories [13], [15], [25]. There is thus a need for a more facile and less costly molecular method to overcome the drawbacks of the current commercial molecular methods.

The fecal sample matrix represents a challenge to sample preparation for molecular methods due to its high inhibitor content. Inhibitors found in stool that are known to affect both PCR and isothermal methods, such as LAMP, include polyphenols [27]–[28], divalent cations [29], acidic polysaccharides [30]–[31], hemoglobin/hemin [32]–[33], phytic acid [34] and bile salts [35]. Current molecular tests commonly rely upon multi-step, time consuming silica-based extraction processes [36]. Whilst these methods can be automated, this adds significant cost particularly where the system is a “fully integrated” platform like the GeneXpert: often too expensive for many clinical settings. An alternative approach employed is to dilute the sample by 200–700 fold so as to reduce the concentration of inhibitors; however, such ‘dilutive’ methods significantly reduce test sensitivity. Hence there is a need for a simple, easy to use and inexpensive method that does not compromise test sensitivity.

To address these needs a novel sample preparation method was developed based on the principle of Heat Elution (HE). In this method a fecal sample swab is added to a column containing a buffered cocktail of resins selected to bind fecal inhibitors of nucleic acid amplification. The column is sealed, shaken and then placed into a collection tube having first broken off a bottom seal on the column which allows eluate to exit the column into the collection tube. The column and collection tube is then placed on a heating block at 100°C for 10 minutes. During this incubation, the *C. difficile* cells are lysed and the DNA released into the buffer and fecal inhibitors become bound to the inhibitor removal resins. As the column is heated, pressure inside the column builds which results in the gentle elution of the eluate from the column into the collection tube, leaving the inhibitors bound on the column. The use of the inhibitor removal matrix means that the sample ends up being diluted only 50 fold compared to the 200 to 700 fold dilutions used by dilutive methods. The eluate is then used to directly reconstitute lyophilized LAMP-BART reagent. The robustness of this method also means that both unformed, solid and blood containing stool samples can be tested; this means that it is possible to test for *C. difficile* carriers who are not presenting with symptoms of diarrhea.

The HE method is described herein in conjunction with Loop-mediated Isothermal Amplification (LAMP) employing the BART (bioluminescent assay in real-time) bioluminescent reporter system. The BART reporter system is designed to work with Isothermal Nucleic Acid Amplification Technologies (iNAATs) and has the advantage over fluorescent and turbidity reporter systems of requiring exceptionally simple, robust and low-cost hardware. The LAMP-BART combination has been well characterized, is robust to inhibitors and forms the basis of commercially available molecular test kits [37]–[39].

This article describes a pilot study of the Heat Elution method in combination with a LAMP-BART test for the toxin B gene of *C. difficile* (*tcdB* ^+^). HE-LAMP-BART was compared to cytotoxigenic culture done when the samples were obtained and a PCR method developed by Public Health England (PHE) to control for sample degradation with storage. The PHE PCR was a home-brew multiplex PCR method using the easyMAG extraction method from bioMérieux that was modified by the Clinical Microbiology Laboratory at Addenbrooke’s Hospital (PHE), Cambridge, UK for stool extraction.

The test was also compared against a number of commercial tests, including the Illumigene, GeneXpert and RIDAQuick tests. In addition to the comparative performance on clinical stool specimens, we have also described the analytical sensitivity, performance with interferents as well as the inclusivity and exclusivity of the HE-LAMP-BART *C. difficile* test.

## Materials and Methods

### Requirement of ethical approval

It was confirmed by the Research Ethics Chair of Cambridgeshire 2 Research Ethics Committee that ethical approval was not required for the study because it was an evaluation of an established nucleic acid amplification technology that did not involve the use of linked samples, access to patient details, any additional sample other than that required for routine *C. difficile* screening nor would it screen for additional pathogens other than toxigenic *C. difficile*.

### Bacterial strains and culturing

The following *C. difficile* strains were obtained from the National Collection of Type Cultures: NCTC 13307, NCTC 11205, NCTC 11209 and NCTC 11204. Non-toxigenic *C. difficile* strain ATCC 43593 was obtained from the American Type Culture Collection. Other organisms used were from an in-house Lumora culture collection. *C. difficile* strains were cultured anaerobically on blood agar (Oxoid), Brazier’s agar (Oxoid) or in brain heart infusion (Difco). Other organisms were cultured in tryptone soya agar (Oxoid) or buffered peptone water broth (Oxoid).

### Stool specimens

Stool specimens sent to PHE Microbiology services-Cambridge from the East of England region were collected over a 17 month period (July 2010 to November 2011) and stored at -20°C. For this evaluation study we sought to include at least 100 known *C. difficile* positive stool specimens balanced with around the same number of negative specimens. Such a sample size would provide a 95% confidence interval of +/– 5.9% for an estimated test sensitivity (or specificity) of 90%, which is in line with recommended guidelines [40]. Of 218 stool specimens selected consecutively over the study period with sufficient volume to permit multiple testing, 196 were unformed and 22 were solid. The status of these samples had already been determined by VIDAS® *Clostridium difficile* A & B (bioMérieux, FR), toxigenic cell culture assay, culture and ribotyping, and comprised 111 *C. difficile* toxin positives and 107 negatives, and provided the gold standard reference test result for benchmarking. A broad range of different ribotypes were observed in the positive specimen group and are detailed below. Specimens were assigned numbers unique to the study and were blinded and aliquoted for the study.

### DNA standards

Genomic DNA from *C. difficile* 630 was either a kind gift from Dr. Trevor Lawley at the Wellcome Trust Sanger Institute, Cambridge or purchased from ATCC.

### Heat Elution extraction & LAMP-BART

**Figure 1:**
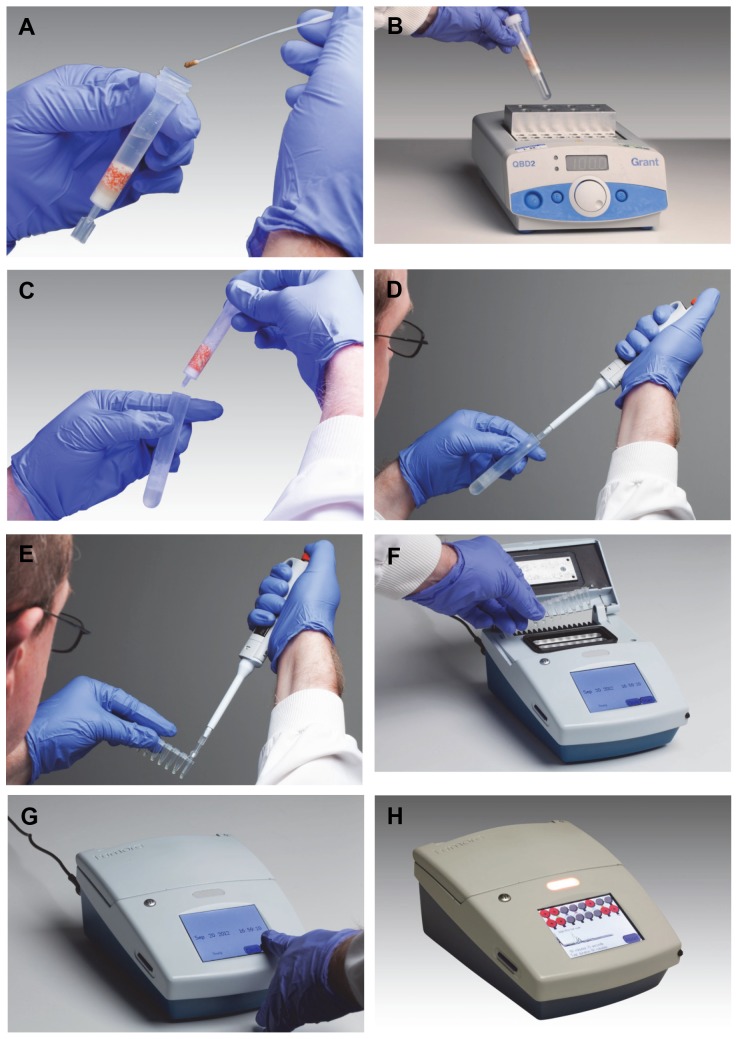
Photographic illustration of the steps of the HE-LAMP-BART. A. Place fecal swab sample into the HE column, mix, seal and twist off the bottom tab of the column; B. Place the column in a collection tube on a hot block at 100°C and incubate for 10 minutes; C. Remove the collection tube and dispose of the column; D&E. Pipette 20 µl of eluate from the collection tube and use this to reconstitute LAMP-BART freeze dried reagent; F&G. Place reconstituted reagent on PDQ and start run; H. Positive results are called in real time. The person in the photograph has given written informed consent, as outlined in the PLOS consent form, to publication of their photograph.

Heat Elution extraction was performed as follows (Figure 1). Each stool sample was swabbed with a micro ultrafine tipped flocked swab (Puritan, Guilford, ME). The swab was placed in the HE column and mixed by rotating the swab between the fingers. The shaft of the swab was then broken at the breakpoint and the swab sealed inside the column by putting the cap on the column and the sample then mixed in the column by gently shaking. The twist-off tab at the bottom of the column was then snapped off to open the column, which was placed into a collection tube and placed on a heat block at 100°C and incubated for 10 minutes. During this time inhibitors are bound to the inhibitor removal matrix cocktail, cells lysis occurs and liberated DNA is gently eluted in lysis/amplification buffer into the collection tube. The collection tube and column was then removed from the hot block and the column discarded. When cool, 20 µl of eluate from the collection tube was used to reconstitute lyophilized *C. difficile* LAMP-BART reagent. A further 20 µl of eluate was used to reconstitute Inhibitor Control LAMP-BART reagent. This reagent contains both primers and associated 10^4^ copies of an artificial DNA sequence which has been well characterized for the effect of inhibitors on amplification. These reactions were then loaded onto a 16 well photodiode-based reader from Lumora (known as a PDQ). Data was collected over 90 minutes for each amplification. On the PDQ, light output from the reactions is displayed and positive results are called by an algorithm that looks for peaks in real time. Negative or undetermined results are called by the algorithm at the end of the run. This algorithm did not change over the course of the study and all peak results were confirmed by visual inspection. Each run carried a positive control of *C. difficile* genomic 630 DNA (ATCC) in lysis/amplification buffer and a negative control of just lysis/amplification buffer.

### easyMAG extraction and PHE multiplexed real time PCR

Samples of faecal materials were taken using regular flocked swabs (microRheologics; REF 520CS01). Swabs were broken off into screw-cap Eppendorf tubes containing 1 ml 1x Tris-EDTA (TE) buffer (Becton Dickinson) and vortexed at full speed for 10 s. 500 µl of the resultant suspensions were transferred to lysis tubes (Becton Dickinson) containing glass beads (425–600 µm) and vortexed at full speed for 5 mins. 200 µl of the resultant lysates were added to 2 ml lysis buffer (bioMérieux) and nucleic acids extracted using the Generic 2.0.1 protocol with ‘off-board’ lysis on the easyMAG instrument (bioMérieux). Purified nucleic acids were eluted in 85 µl, of which 5 µl were used as template in 25 µl real-time PCR reactions.

PCR assays contained: 5 µl of nucleic acid sample, 12.5 µl of 2x Platinum Quantitative PCR SuperMix-UDG (Invitrogen), 0.5 µl of MgCl_2_ (50 mM), 1.0 µl of Internal control oligonucleotide, each of the corresponding forward and reverse primers and corresponding probes at the concentrations given in Table 1, and DNase-free water (Invitrogen), giving a final volume of 25 µl. All primers and probes were purchased from Metabion, Munich, Germany, with the exception of the two minor groove binding (MGB) Taqman® probes which were purchased from Applied Biosystems, Warrington, UK (Sequences are described in Table 1).

**Table 1:** Primers and probes used for real-time PCR.

Target	Sequence (5’ → 3’)	Function (concentration)	Product size	Primer/Probe source
*tcdB*	CCAAARTGGAGTGTTACAAACAGGTGTA	Forward primer (800 nM)	99 bp	This Study
	GCTTCICCYTCTAGRTTTTCATCAAGTGTA	Reverse primer (400 nM)		
				
	VIC – CCATCTTCTGTACTAA - MGB	Probe (80 nM)		
GDH	GTAATACCAGCTGCATTAGAAAACTC	Forward primer (400 nM)	89 bp	This Study
	GGTCCATTAGCAGCCTCACAA	Reverse primer (400 nM)		
				
	6FAM – TAGATTCAGCAACTTC - MGB	Probe (80 nM)		
Internal	CTCTGCTTTATATTATAAAATTACGGCTG	Forward primer (400 nM)	104 bp	This Study
Control	ACTTTAGTCAAATCATCTTCACTAGTG	Reverse primer (400 nM)		
(IC)				
	ROX –	Probe (100 nM)		
	CACATCGATAGATCAAGGTGCCTACAAGC -			
	BHQ2			
IC	CTCTGCTTTATATTATAAAATTACGGCTGGGCGTTAAGTGTCACATCGATAGATCAAGGTGCCTACAAGCGAAGTGGCACTAGTGAAGATGATTTGACTAAAGT			This Study

A specially designed Internal control oligonucleotide DNA (approximately 1000 copies per reaction) was added to real-time PCR reactions as a control for PCR inhibition (Table 1). The control was detected in multiplex real-time PCR reactions concomitantly with the target pathogen nucleic acids. Real-time PCR assays were performed using a Rotor-Gene Q (Qiagen), according to the following profile: 50°C for 120 s, 95°C for 120 s, and then 45 cycles of 95°C for 10 s and 60°C for 60 s. Fluorescence data acquisition was performed at the end of each 60°C incubation period.

The PHE in-house real-time PCR assay was used in this study to provide a direct comparator NAAT for LAMP-BART ensuring any specimen degradation during storage did not unfairly bias the evaluation. While the in-house PCR assay is not used routinely in our diagnostic service (PHE) its performance has been fully assessed and validated. Briefly, 90 consecutive stool specimens sent for *C. difficile* testing to our laboratory were also processed on the GeneXpert system and our in-house real-time assay. Complete concordance was observed between the two methods, with 17 *C. difficile* toxigenic positive stools and 73 negative stools, which was also in agreement with our routine diagnostic results. A range of *C. difficile* isolates with different ribotypes (001, 002, 003, 005, 012, 014, 015, 016, 017, 020, 023, 026, 027, 029, 046, 050, 072, 078, 081, 106, 107, 137, 174) were all detected by the PHE RT PCR assay and it passed a specificity PCR check with a comprehensive bank of chromosomal DNA extracted from bacterial organisms known to populate the intestinal flora. The performance is also assessed every year using an external quality assessment panel obtained from Quality Control for Molecular Diagnostics (www.qcmd.org). A 100% score has been achieved for the last three years (2010, 2011 and 2012) with their QCMD *Clostridium difficle* DNA EQA Programme (10 samples in each EQA panel).

### Illumigene *C. difficile* LAMP assay

Illumigene *C. difficile* LAMP assay (Meridian, Cincinnati, OH) was performed on 75 stool samples selected from the PHE multiplex RT-PCR/HE-LAMP-BART method comparison. These particular samples were selected at Lumora as being especially challenging due to either low copy number as indicated by high PHE Ct value, high inhibitor load by delayed inhibitor control peak, coming from solid stool, blood content and less common ribotype. The IllumiPro-10 instrument was used as described by the manufacturer with the exception that formed stools were tested to assess the test’s ability to detect *C. difficile* in formed stool. Samples were run at the same time on both the Illumigene and HE-LAMP-BART methods. Positive and negative controls were run on the test once a day.

### Xpert *C. difficile*/EPI PCR assay

The Xpert *C. difficile*/EPI PCR assay (Cepheid, Sunnyvale, CA) was performed on 27 stool samples further selected from the Illumigene/LAMP-BART method comparison for low copy number, high inhibitor load, blood content and less common ribotype. The GeneXpert *C. difficile* cartridge was used as described by the manufacturer with the exception that formed stools were tested to assess the test’s ability to detect *C. difficile* in formed stool.

### RIDA®QUICK *Clostridium difficile* Toxin A/B rapid test immunoassay

RIDA®QUICK *Clostridium difficile* Toxin A/B rapid test (R-Biopharm, Darmstadt, Germany) was performed on the same 27 stool samples used for the GeneXpert comparison as described by the manufacturer.

### Interference study for HE-LAMP-BART

Interfering substances likely to be found in stool samples were purchased either from Sigma (Poole, UK) or from retail/pharmacy. Clinically relevant amounts of these substances were added to a negative stool sample spiked with 32 CFU/ reaction equivalent of *C. difficile* NCTC 13307 extracted by Heat Elution and the eluate run in *C. difficile* and Inhibitor Control LAMP-BART reagent.

### Stool spike detection comparison

A *C. difficile*-negative unformed stool sample was spiked with 10,000 CFU/ml *C. difficile* NCTC 13307 and a two-fold dilution series down to 312.5 CFU/ml made using the same negative stool sample. Each level was tested by HE-LAMP-BART, PHE easyMAG plus RT-PCR, GeneXpert, Illumigene and RIDAQuick.

### HE-LAMP-BART stool spike Limit of Detection

Twenty replicates of eluate from HE extract of *C. difficile* negative unformed stool sample spiked with 8 CFU/reaction equivalent *C. difficile* NCTC 13307 were tested using LAMP-BART.

### Comparative analytical sensitivity between LAMP-BART and PHE RT-PCR

A dilution series of *C. difficile* 630 chromosomal DNA (Sanger Institute, UK) in 10 fold steps down to 100 copies and two fold steps down to 0.5 copies were tested by both LAMP-BART and PHE RT-PCR.

### Inclusivity and exclusivity of the *C. difficile* LAMP-BART test

The inclusivity of the *C. difficile* LAMP-BART assay was evaluated by testing a panel of 5 *C. difficile* strains; NCTC 13307, NCTC 11205, NCTC 11209, NCTC 11204 and the non-toxigenic strain ATCC 43593. Bacteria were grown on blood agar plates and single colonies diluted with vortex mixing in the equivalent of 10 ml of Buffered Peptone Water followed by a 90-fold dilution in reaction buffer. The final reaction buffer dilutions were heated at 110°C for 5 minutes. When cool, lysates were tested in singlet with the Lumora *C. difficile* LAMP-BART assay. In the same way, the exclusivity of the *C. difficile* LAMP-BART assays was evaluated by testing a panel of 37 bacteria. Bacteria were either grown on TSA or blood agar plates and single colonies, or the equivalent, were transferred to 250 µl of reaction buffer, vortex mixed and heated at 110°C for 5 minutes. When cool, lysates were tested in duplicate with the Lumora *C. difficile* LAMP-BART assay. The following exclusive organisms were tested with the *C. difficile* LAMP-BART assay: *Aeromonas hydrophilia*, *Bacillus cereus*, *Campylobacter jejuni*, *Citrobacter amalonaticus*, *Citrobacter freundii*, *Clostridium perfringens*, *Clostridium sordellii*, *Edwardsiella tarda*, *Enterobacter aerogenes*, *Enterobacter cloacae*, *Enterococcus gallinarum*, *Escherichia coli*, *Hafnia alvei*, *Klebsiella oxytoca*, *Klebsiella pneumoniae*, *Lactobacillus acidophilus*, *Listeria grayii*, *Listeria innocua*, *Listeria ivanovii*, *Listeria monocytogenes*, *Listeria seeligeri*, *Proteus mirabilis*, *Proteus vulgaris*, *Pseudomonas aeruginosa*, *Pseudomonas fluorescens*, *Pseudomonas putida*, *Salmonella enteritidis*, *Salmonella typhimurium*, *Serratia liquefaciens*, *Shigella sonnei*, *Staphylococcus aureus*, *Staphylococcus epidermidis*, *Streptococcus agalactiae*, *Vibrio parahaemolyticus* and *Yersinia enterocolitica*.

### Statistical analysis

Sensitivity/specificity and confidence intervals were determined using StatsDirect Statistical Software version 2.7.9 (http://www.statsdirect.com).

## Results

### Typical HE-LAMP-BART results

**Figure 2:**
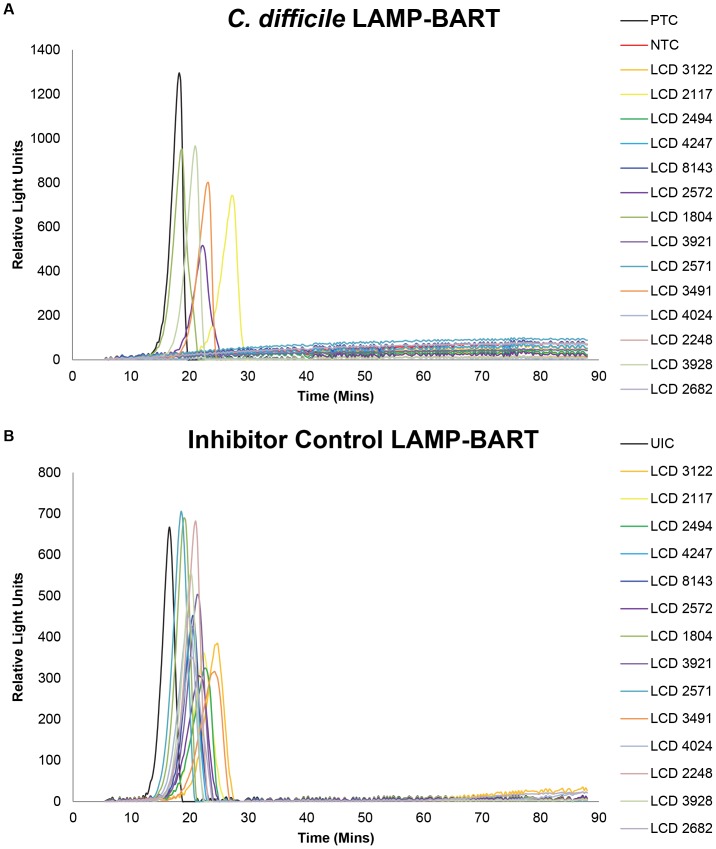
Example *C. difficile* HE-LAMP-BART results. One HE-LAMP-BART run of 14 samples from the Illumigene comparison. Panel A shows the *C. difficile* LAMP-BART peaks obtained and includes a positive control of 10^4^ copies of *C. difficile* genomic DNA (PTC) and a no template control (NTC). Panel B shows the Inhibitor Control LAMP-BART results obtained simultaneously from the same set of HE eluates and a buffer uninhibited control (UIC).

The LAMP-BART results from one of the runs from the comparison with Illumigene are presented in Figure 2 and Table 2. In addition to the *C. difficile* reactions, the Inhibitor Control results are also displayed. If the Inhibitor Control peak time was later than 35 minutes a retest was necessary if the *C. difficile* test for that sample was negative. The HE-LAMP-BART is a two-step process that takes typically 30 to 60 minutes from swabbing the stool sample to obtaining a positive peak on the instrument. Within the Illumigene comparison the average LAMP-BART time to result was 37±6.0 minutes.

**Table 2:** Example *C. difficile* HE-LAMP-BART results.

	*C. difficile*		Inhibitor Control	
Sample	Peak Time (min)	Result	Peak Time (min)	Result
PTC	15.5	POS		
NTC		NEG		
UIC			13.75	POS
LCD 3122		NEG	21.5	POS
LCD 2117	24	POS	19.5	POS
LCD 2494		NEG	19.5	POS
LCD 4247		NEG	17.5	POS
LCD 8143		NEG	17.75	POS
LCD 2572	19.5	POS	18.25	POS
LCD 1804	15.75	POS	16.25	POS
LCD 3921		NEG	18.25	POS
LCD 2571		NEG	15.75	POS
LCD 3491	20	POS	20.25	POS
LCD 4024		NEG	16.75	POS
LCD 2248		NEG	18	POS
LCD 3928	18	POS	17.25	POS
LCD 2682		NEG	17.25	POS

Peak time and results called for the example LAMP-BART data set presented in Figure 1. For the *C. difficile* assay Positive control of 10^4^ copies of *C. difficile* genomic DNA (PTC) and a no template control (NTC) were used. For the inhibitor control LAMP-BART results a buffer uninhibited control (UIC) was used.

### Comparative analytical sensitivity between LAMP-BART and PHE RT-PCR

Both the PHE RT-PCR and LAMP-BART tests reproducibly detected down to 10 copies per amplification reaction of *C. difficile* 630 genomic DNA in quadruplicate.

### Stool spike sensitivity comparison

In this experiment GeneXpert and HE-LAMP-BART gave complete detection down to 5000 CFU/ml, Illumigene detected down to 2500 CFU/ml and PHE RT-PCR detected down to 312.5 CFU/ml. None of the spike levels tested were detected by RIDAQuick Toxin A/B rapid test immunoassay. Although we do not fully understand this result, it could be due to the toxins not being expressed by cultured *C. difficile* [21] or the dilution of expressed toxin proteins.

### Inclusivity and exclusivity of LAMP-BART

*C. difficile* strains NCTC 13307, NCTC 11205, NCTC 11209, NCTC 11204 were detected by the HE-LAMP-BART assay. Non-toxigenic strain ATCC 43593 was not detected and none of the exclusive organisms tested were detected. In addition, from the fecal samples, 78 of the 111 *tcdB* harboring strains with ribotypes 001, 002, 003, 005, 011, 012, 013, 014, 015, 020, 023, 026, 027, 046, 050, 078, 081, 126, 150, 258, 351 and 355 were successfully detected by HE-LAMP-BART. The remaining 33 positive isolates were also detected, but reported as sporadic (with respect to their ribotype) as their ribotypes did not match any of the ribotype profiles currently held in our laboratory database.

### Interferents

The concentrations of interferents shown in Table 3 did not interfere with the detection of 32 CFU/reaction of *C. difficile* NCTC 13307 spiked into a *C. difficile* negative unformed stool sample.

**Table 3:** Concentrations of substances that do not interfere with the detection of *C. difficile* by HE-LAMP-BART.

Interferent	Conc. in stool
Anusol	40%
Gygel	40%
1% Hydrocortisone	40%
KY Jelly	40%
Peptobismol	40%
Vaseline	40%
Preparation H	40%
Stearic acid	20%
Palmitic acid	20%
Haemoglobin	20%
CaCO_3_ Tabs	10%
Lactulose	10%
Senokot	10%
Benzalkonium Cl	10%
Barium sulphate	5%
Milk of Magnesia	5%
Micoconazole	20 mg/ml
Phenylephrine	20 mg/ml
Metronidazole	10 mg/ml
Vancomycin	10 mg/ml
Imodium	10 mg/ml
Nystatin	10000 USP u/ml
Mucin	3.5 mg/ml
Feminax Ultra	1.25 mg/ml

### Performance on Clinical Stool Samples

**Method comparison between HE-LAMP-BART with gold standard reference & PHE multiplex RT-PCR.** A total of 218 stool samples were tested by HE-LAMP-BART *C. difficile* and compared to the cytotoxic culture reference method, with sample status after -20°C storage confirmed by PHE multiplex RT-PCR following easyMAG extraction (Table 4). 106 out of 111 *C. difficile* positive samples were positive by HE-LAMP-BART resulting in 5 false negatives and 95.5% sensitivity. HE-LAMP-BART was concordant with the reference and PHE methods for negative samples. Thus, HE-LAMP-BART gave very good agreement with the cytotoxigenic culture reference.

**Table 4:** Comparison between the reference method and PHE-Cambridge multiplex RT-PCR and HE-LAMP-BART.

		Total	PHE RT-PCR Positive	PHE RT-PCR Negative	HE-LAMP-BART Positive	HE-LAMP-BART Negative
**PHE**	**Total**	218	106	112	106	112
**Reference**	**Positive**	111	111	0	106	5
**Test/method**	**Negative**	107	0	107	0	107
**Sensitivity (95% CI)**			100% (96.73–100%)		95.5% (89.8–98.52%)	
**Specificity (95% CI)**			100% (96.61–100%)		100% (96.61–100%)	

**Comparison between HE-LAMP-BART and the Illumigene LAMP Methods.** On the subset of 75 challenging stool samples (including samples with low copy number, solid stool, samples with high inhibitor load, containing blood and less common ribotype), compared to the cytotoxic culture reference method, PHE RT-PCR gave 100% sensitivity, HE-LAMP-BART gave 87.2% sensitivity compared to 76.6% sensitivity for Illumigene (Table 5) due to 6 and 11 false negatives, respectively. The Illumigene method gave one consistent invalid result for a negative sample with high blood content. Only one sample was detected as *C. difficile* positive by Illumigene, but not by HE-LAMP-BART; whereas 6 samples were detected as *C. difficile* positive by HE-LAMP-BART, but negative by Illumigene. However, when the former Illumigene positive/HE-LAMP-BART negative sample was re-extracted and run with *C. difficile* LAMP-BART in sextuplet, 4 out of the 6 reactions were detected as positive, indicating a low copy number sample close to the sensitivity of the HE-LAMP-BART method. These sensitivity values should be considered in the context of the challenging sample subset tested. All tests showed 100% specificity.

**Table 5:** Comparison of HE-LAMP-BART and the Illumigene LAMP Methods against the reference method.

	Illumigene	HE-LAMP-BART	PHE Reference Test/Method
Positives	36	41	47
Negatives	38	34	28
Invalid	1	0	
Total	75	75	
False Positives	0	0	
False Negatives	11	6	
Sensitivity (95% CI)	76.6% (61.97–87.7%)	87.2% (74.26–95.17%)	
Specificity (95% CI)	100% (87.66–100%)	100% (87.66–100%)	

**Extension of comparison to a subset from the Illumigene-HE-LAMP-BART study to GeneXpert and RIDAQuick.** A further subset of 27 challenging stool samples, including low copy number, high inhibitor load, blood containing and less common ribotype samples from the HE-LAMP-BART vs Illumigene study were also tested by the GeneXpert and RIDAQuick methods. Compared to the cytotoxic culture reference method and PHE RT-PCR results, the following sensitivities were obtained: GeneXpert 96%, HE-LAMP-BART 92%, Illumigene 72% and RIDAQuick 76% (Table 6) due to 1, 2, 7 and 6 false negatives, respectively. For the RIDA Quick Toxin A/B method, 6 out 19 positive results gave weak positive test lines. Again, these sensitivity values should be considered in the context of the challenging sample subset tested and all tests showed 100% specificity.

**Table 6:** Comparison of a subset of samples from the Illumigene-HE-LAMP-BART study with GeneXpert and RIDAQuick against the reference method.

	PHE Reference Test/Method	PHE RT-PCR	GeneXpert	HE-LAMP-BART	Illumigene	RIDA Quick Toxin A/B
Positive	25	25	24	23	18	19
Negative	2	2	2	2	1	2
Total	27	27	27	27	27	27
False Positive		0	0	0	0	0
False Negative		0	1	2	7	6
Invalid		0	0	0	1	0
Sensitivity	%	100	96	92	72	76
	%CI	86.28–100	79.65–99.9	73.97–99.02	50.61–87.93	54.87–90.64
Specificity	%	100	100	100	100	100
	%CI	15.81–100	15.81–100	15.81–100	15.81–100	15.81–100

## Discussion

DNA preparation for current commercial molecular tests for *C. difficile* in stool either rely on robotic extraction that is integral to the test (GeneXpert), robotic or manual Boom style extraction before the test or a multistep manual dilutive sample preparation method involving a 200–700 fold dilution to overcome inhibition by diluting the fecal inhibitors (Illumigene).

The GeneXpert test from Cepheid uses a 2 addition sample preparation procedure consisting of swabbing the stool sample, adding the swab to a small bottle containing Sample Reagent, which is then vortexed and the contents then transferred by pipette to the cartridge. The cartridge is then loaded on the GeneXpert Dx instrument where sample extraction and PCR take place over 45 minutes resulting in detection of most positive samples reported in real time at about 30 minutes. It has been reported that GeneXpert requires 4 minutes technical time and a turnaround time of 45 minutes [41]. The complicated GeneXpert Dx instrument GeneXpert Dx that performs extraction, PCR cycling and fluorescence detection, could therefore be too expensive for certain clinical settings.

The Illumigene test from Meridian uses a 7 step, 4 transfer procedure between specimen collection with a Sample Collection Brush to reconstituting both test and control lyophilized beads reagent. These reaction tubes are transferred to the Illumipro-10 and amplified for 40 minutes with the result being displayed at the end of the run. Illumigene takes 5 minutes, has a turnaround time of 1 hour and does not require costly capital equipment [41].

The HE-LAMP-BART procedure, as described above, has the same number of steps as GeneXpert, has less hands-on time than the Illumigene sample preparation and can report the majority of positive samples within 45 minutes of swabbing. The photodiode PDQ instrument itself is small, has a low-cost of goods and is exceptionally robust.

In the performance comparison between the PHE real time *C. difficile* PCR and HE-LAMP-BART against the cytotoxigenic culture reference method, the PHE method showed perfect detection of toxigenic *C. difficile*. Although the PHE method costs $45 per specimen and has significant hands on time for extraction and set up of the PCR assay. HE-LAMP-BART showed very good agreement with the cytotoxigenic reference in detecting 95.5% of the positive samples. The comparison between Illumigene and HE-LAMP-BART on a challenging subset showed that the HE-LAMP-BART method had a sensitivity of 87.2% compared to 76.6% for the Illumigene method. The Illumigene turbidimetric detection method suffered when compared to HE-LAMP-BART in the detection of a blood rich sample. Illumigene has already been reported as giving invalid results in blood rich samples [41]–[42]. In this sample we observed a blood derived particulate in the reaction buffer that would be expected to interfere with detection. HE-LAMP-BART was not inhibited by this blood rich sample and the hemoglobin in the interference study, which already exhibited a strong inhibitor tolerance, showed that a stool sample containing 20% hemoglobin would still be detected. Whilst the GeneXpert method is able to tolerate fecal blood, it is suggested in the kit instructions that fecal particulates can interfere with the method. Due to the design of the Heat Elution columns, such particulates do not interfere with the HE-LAMP-BART method.

**Figure 3:**
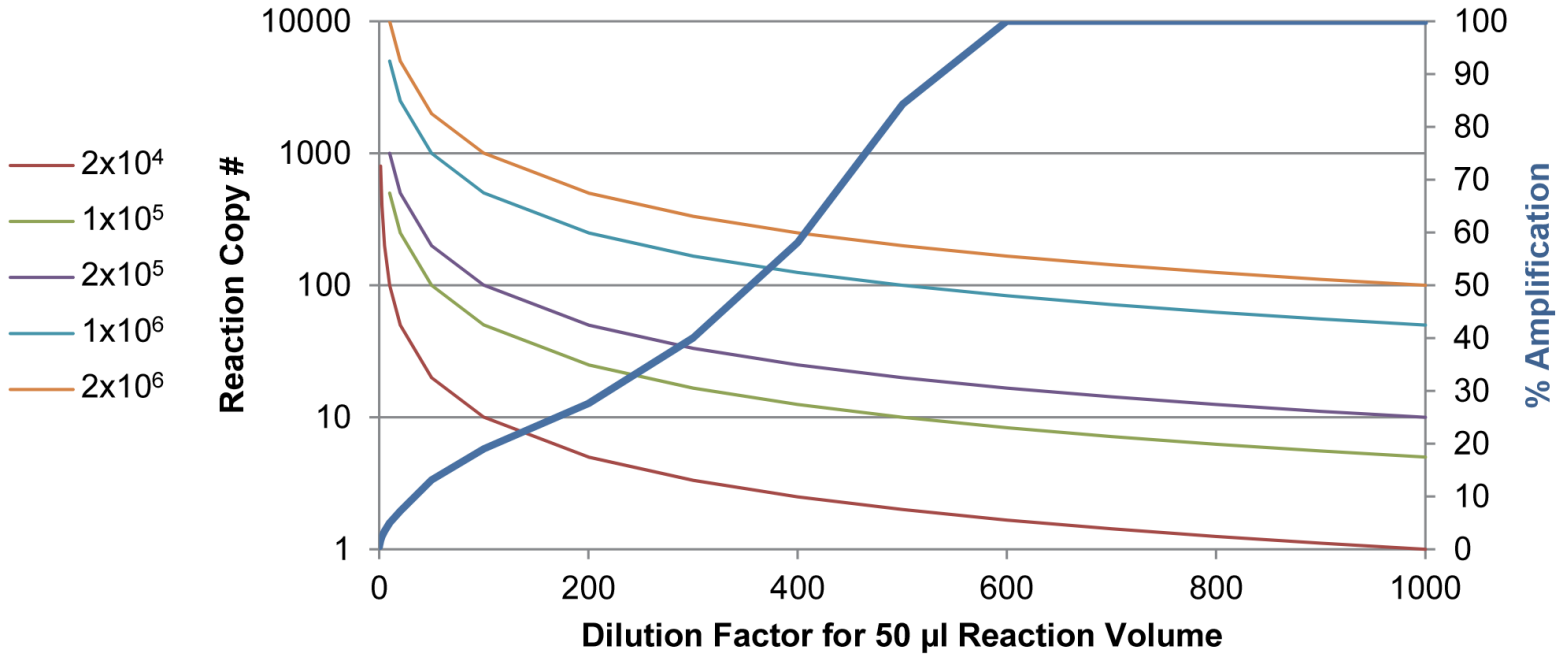
Model describing low copy number non-detection for dilutive methods. The right hand Y-axis (% amplification) shows the increase in amplification associated with dilution of inhibitors from a fecal sample. A range of starting target concentrations (CFU/gram) is plotted with the left hand Y-axis showing the copy number present in a 50 µl reaction volume (Illumigene). The reduced inhibition and increasing % amplification with increasing dilution is concomitant with a reduction in copy number. At lower CFU/gram samples, the dilution places the copy number per reaction beneath the sensitivity of the assay. HE-LAMP-BART used a 50 fold dilution together with the inhibitor removal such that a higher copy number can be present in the reaction.

In the smaller study comparing the HE-LAMP-BART results with GeneXpert, RIDAQuick and Illumigene, GeneXpert showed a sensitivity of 96%, followed by HE-LAMP-BART at 92%, RIDAQuick at 76% and Illumigene at 72%. GeneXpert had already been reported to show superior sensitivity to Illumigene [41]. It was unexpected that the RIDAQuick immunoassay showed a higher sensitivity than Illumigene. To understand this we carefully examined all the data for these samples by all methods. Most of these samples displayed late PHE Ct values that became further delayed or failed when given a further 10-fold dilution. We observed a similar pattern by diluting some of these fecal samples by 200, 500 and 700 fold (factors typical of commercial dilutive methods) and running heat lysates on *C. difficile* LAMP-BART. This suggested that these samples had a low number of *C. difficile* that when subjected to the Illumigene dilutive method still had inhibitor at a concentration sufficient to slow down or stop amplification such that it could not be detected within the 40 minute amplification time. We have arrived at a model to explain the dilutive method low copy number failure. According to this model, illustrated in Figure 3, in the absence of inhibitor a reaction can show 100% amplification efficiency. At the highest concentration of inhibitor no amplification is seen; but as the fecal sample is given a gradual dilution, a gradual increase in amplification efficiency can occur up to over 700 fold for stool when inhibition becomes negligible. On the other hand, as this dilution proceeds, the DNA copy number also gradually decreases, such that samples of very low *C. difficile* content will have a very low DNA copy number in the assay. We have determined that the Illumigene assay uses a 300 to 400-fold dilution depending on the volume range of the sample swabbed. In comparison to 500-fold dilution and above, a 300 to 400-fold dilution leaves a sufficient inhibitor concentration to inhibit amplification of a, *e.g.* 2×10^4^ CFU/gram, sample diluted to assay sensitivity level. In contrast, the inhibitor removal by the Heat Elution process provides an eluate diluted only by 50-fold that can contain 6-8 times more concentrated target due to the lower dilution factor. Thus, the difference in sensitivity of HE-LAMP-BART (87.2%) over Illumigene (76.6%) may have been a result of the lower dilution factor combined with robust inhibitor removal in HE-LAMP-BART. In addition to the use of Heat Elution for LAMP-BART, we have also tested eluate in real time PCR reactions, without any optimization, and had promising results showing detection of all high copy number samples (data not shown). Furthermore, we anticipate that the method would be applicable to other bacterial diarrhea pathogens.

The observation above is in agreement with the observation of Viala *et al*. that some commercial PCR tests reporting false negatives have been attributed to target copy numbers below the sensitivity limits of the tests [43]. When low copy number HE eluates were run in *C. difficile* LAMP-BART (in sextuplet), positive detection was seen in typically ≤4 out of the 6 replicates. From this result it is clear to see how such low copy number samples with DNA content around the sensitivity limit of the assay could show variable detection when single replicates are run.

Based on testing with spiked fecal samples, Bélanger *et al*. [44] showed that their cycle thresholds range of 22 to 35 corresponded to 10^7^ to 10^4^ CFU/gram. They further suggested that Ct 35 patients could have been *C. difficile* carriers exhibiting diarrheal symptoms of another etiology. A number of studies have reported asymptomatic adult carriage of *C. difficile* in the range 0.5–13% [45]–[47]. This increases to up to 14% in hospitalized patients [48]–[50] and is as high as 52% in long term care facilities [50]–[51]. A study by de Jong *et al*. [45] concluded that neither PCR nor cytotoxic culture is able to distinguish between CDI and asymptomatic carriage. It has also been suggested that such asymptomatic carriers contribute to the transmission of CDI [50]. However, it seems plausible that samples sub-detectable by HE-LAMP-BART, and indeed Illumigene, may not have been CDI cases; although for ethical reasons this study has not had access to patient information to prove this. A full clinical trial is needed to determine the performance of the HE-LAMP-BART method on stool samples from diagnosed CDI. Compared to one of the latest reports using the Boom method, our method is much simpler [52]. The HE-LAMP-BART method described here is a rapid, facile, real time molecular method with a low cost of goods for the detection of *C. difficile* that lends itself to use in decentralized environments.
